# Bioprocess automation on a Mini Pilot Plant enables fast quantitative microbial phenotyping

**DOI:** 10.1186/s12934-015-0216-6

**Published:** 2015-03-11

**Authors:** Simon Unthan, Andreas Radek, Wolfgang Wiechert, Marco Oldiges, Stephan Noack

**Affiliations:** Institute of Bio- and Geosciences, IBG-1: Biotechnology, Systems Biotechnology, Forschungszentrum Jülich, 52425 Jülich, Germany

**Keywords:** Mini Pilot Plant, Bioprocess automation, Microtiter plate assay, Scale-up, Scale-down, High-throughput, Screening, Microbial phenotyping, D-xylose, Ninhydrin, L-lysine, *C. glutamicum*

## Abstract

**Background:**

The throughput of cultivation experiments in bioprocess development has drastically increased in recent years due to the availability of sophisticated microliter scale cultivation devices. However, as these devices still require time-consuming manual work, the bottleneck was merely shifted to media preparation, inoculation and finally the analyses of cultivation samples. A first step towards solving these issues was undertaken in our former study by embedding a BioLector in a robotic workstation. This workstation already allowed for the optimization of heterologous protein production processes, but remained limited when aiming for the characterization of small molecule producer strains. In this work, we extended our workstation to a versatile Mini Pilot Plant (MPP) by integrating further robotic workflows and microtiter plate assays that now enable a fast and accurate phenotyping of a broad range of microbial production hosts.

**Results:**

A fully automated harvest procedure was established, which repeatedly samples up to 48 wells from BioLector cultivations in response to individually defined trigger conditions. The samples are automatically clarified by centrifugation and finally frozen for subsequent analyses. Sensitive metabolite assays in 384-well plate scale were integrated on the MPP for the direct determination of substrate uptake (specifically D-glucose and D-xylose) and product formation (specifically amino acids). In a first application, we characterized a set of *Corynebacterium glutamicum* L-lysine producer strains and could rapidly identify a unique strain showing increased L-lysine titers, which was subsequently confirmed in lab-scale bioreactor experiments. In a second study, we analyzed the substrate uptake kinetics of a previously constructed D-xylose-converting *C. glutamicum* strain during cultivation on mixed carbon sources in a fully automated experiment.

**Conclusions:**

The presented MPP is designed to face the challenges typically encountered during early-stage bioprocess development. Especially the bottleneck of sample analyses from fast and parallelized microtiter plate cultivations can be solved using cutting-edge robotic automation. As robotic workstations become increasingly attractive for biotechnological research, we expect our setup to become a template for future bioprocess development.

## Background

Time is the most limiting factor in the initial establishment of new bioprocesses and currently forces process developers to draw conclusions from few experiments with little process insight. Such early decisions are typical for the screening of strain libraries for an improved production strain or during the first coarse-grained characterization of a set of top producer candidates. Clearly, any misinterpretation of the resulting limited data sets can strongly impair the further process development. To solve this dilemma new technological approaches are needed that enable the fast and quantitative assessment of multiple strains under well-defined cultivation conditions.

To this end, sophisticated microliter scale cultivation devices were established, which overcome some limitations considering process insight by online monitoring of biomass, pH and dissolved oxygen as well as the direct determination of oxygen transfer rates [1,2]. However, all these devices still depend on manual work to prepare, sample and finally analyze cultivation experiments and, thus, cannot fully remove the bottleneck of today’s bioprocess development [3].

To overcome these limitations, we already embedded a microtiter plate cultivation device (BioLector) in a robotic liquid handling platform and could show its benefits for the preparation of cultivation media and the optimization of heterologous protein expression [4]. However, no robotic workflow has been presented yet that automatically separates supernatants from biomass in samples of microtiter plate cultivations. Such an at-line clarification would enable the direct analysis of supernatants applying, e.g., sensitive enzyme assays for metabolite quantification. Moreover, it would minimize the reaction of periplasmic or membrane-bound enzymes with extracellular metabolites as well as leakage of intracellular metabolites from cells to the supernatant during sample storage [5]. Current microliter scale cultivations are also not well suited for determining substrate uptake or metabolite production kinetics of an organism as the removal of an adequate sample volume for offline-analytics critically influences the cultivation conditions. For instance, the volume reduction due to sampling immediately changes the oxygen transfer rates in the particular well of a microtiter plate even in cases with less than 10% volume loss [6]. Fortunately, microtiter plate cultivations in a BioLector system offer high well-to-well reproducibility [1] and hence identically inoculated cultures can be started in parallel wells of which one is harvested at a time.

In this work, we extended our robotic approaches to automate complete workflows for the quantitative phenotyping of small molecule producer strains. The resulting Mini Pilot Plant (MPP) now covers a broad range of procedures to automatically harvest, clarify and analyze BioLector cultivation wells after reaching individual triggers. To enable fast analyses of those clarified samples, photometrical assays were developed to instantly quantify substrate and product metabolites in 384-well plate scale. The established fully automated methods were finally applied to address two basic challenges in bioprocess development, namely to identify top producers from a strain library and to investigate the substrate uptake of a novel engineered strain during mixed carbon source cultivation.

## Results and discussion

### Improved triggered sampling of cell-free cultivation supernatants

**Figure 1:**
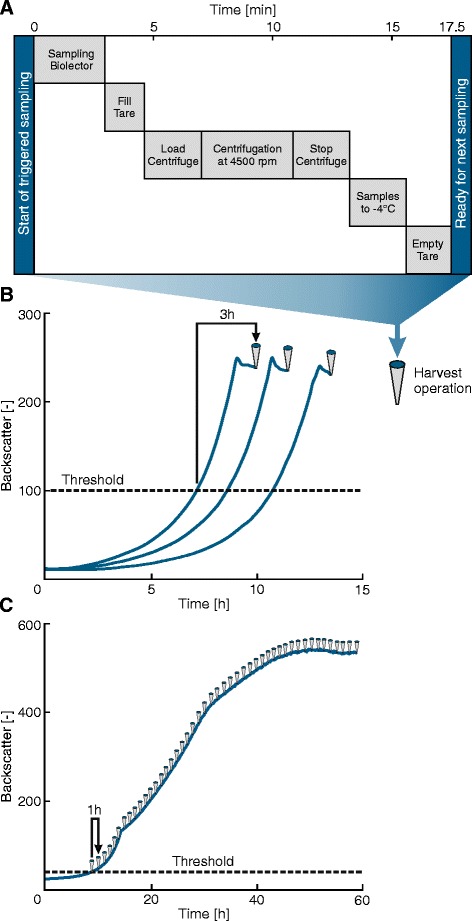
**Fully automated workflow to harvest cell-free cultivation samples from microtiter plate cultivations on the MPP. A**: Steps of the automated harvest operation to generate a variable number (1 - 48) of cell-free supernatants in parallel from BioLector cultivations. **B**: Triggered process to sample supernatants after turn to stationary phase to compare different producer strains considering final product titers. **C**: Time-dependent harvest profile of identically inoculated parallel cultivations to assess metabolite formation or uptake kinetics.

For the screening of protein expression strains, Rohe et al. showed that by transferring cultures to 4°C after their individual turn to stationary phase the amount of produced enzymes could be assessed with higher reproducibility compared to overnight cultivations harvested together at the same timepoint [4]. In this work we further improved the handling of triggered samples on our MPP by introducing additional steps for a rapid centrifugation and freezing to −4°C to generate suitable cell-free samples for subsequent metabolite analyses (Figure 1A).

Our new workflow starts with the harvest of 500 μl from each well of a BioLector cultivation, provided that the individually defined biomass- or time-dependent triggers are reached. Samples are then directly pipetted into a 96 deep-well plate (DWP). After less than 3 minutes interruption, the BioLector closes its lid and continues the cultivation. The time required for the initial sampling depends on the number of wells harvested in parallel, which can vary between 1 and 48 for each cycle. Due to the variable sample number, a second DWP as tare plate for centrifugation is subsequently filled with an equal volume of water and in the same pattern as the sample plate. After following a centrifugation step at 4500 rpm, the resulting cell-free supernatants are transferred to a third DWP at −4°C, the tare DWP is emptied and the MPP is ready for the next sampling event (cf. Figure 1A). The newly introduced rapid cell separation by centrifugation and subsequent freezing of cell-free supernatants diminishes the extent of metabolite leakage, protects heat labile metabolites and prevents the reaction of extracellular enzymes with metabolites during sample storage. One cycle of the developed harvest procedure is finished after 12 to 17.5 minutes for 1 to 48 samples, respectively, and runs fully automated on our MPP without any manual effort.

The established workflow combines the quantitative analysis of a comprehensive set of 48 cultivation experiments wherein different strains under well-defined environmental conditions can be compared with respect to growth and production properties. For example, it can be used to compare the product titers of: (*i*) one production strain cultivated in 48 different media compositions; or (*ii*) a library of 48 different strains cultivated in the same medium; or (*iii*) any reasonable set of 48 combinations thereof (Figure 1B). Furthermore, this workflow can provide insight into the metabolite uptake and production kinetics of an organism when multiple wells of a plate are inoculated with an identical culture and repetitively harvested one after another according to a time-dependent trigger profile (Figure 1C).

### Development and validation of metabolite quantification in microtiter plate scale

To enable the phenotyping of a wide range of amino acid producer strains on our MPP, we developed fast and robust photometric assays for the quantification of amino acids as well as D-glucose and D-xylose in the culture supernatants originating from triggered sampling.

**Figure 2:**
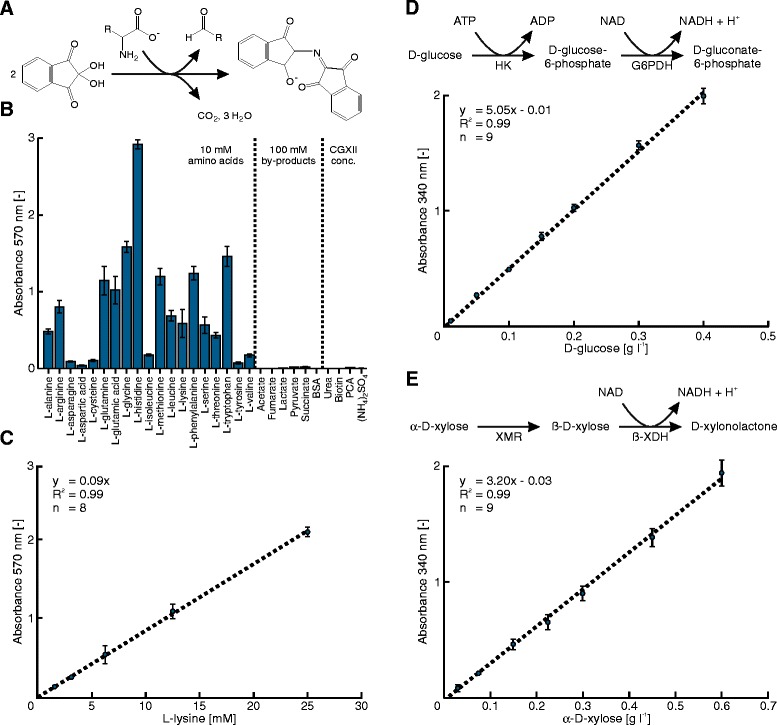
**Development and validation of metabolite quantification in microtiter plate scale on the MPP. A**-**C**: Fast photometric quantification of amino acids was realized by the application of the developed Ninhydrin assay in 384-well microtiter plates. The assay was tested with regard to the measurable metabolite spectrum as well as its selectivity against relevant by-products and media components of the model host *C. glutamicum*. **D**-**E**: Enzymatic assays to quantify D-glucose or D-xylose in culture supernatants were automated in 384-well microtiter plates.

For the determination of amino acid concentrations the well-known Ninhydrin assay [7] was transferred to microtiter plate scale. In short, Ninhydrin reacts with an amino acid to a Schiff base before decarboxylation, dehydration and addition of a second Ninhydrin molecule leads to the formation of the Ruhemann’s purple (Figure 2A). During the transfer of the established Ninhydrin protocols to microtiter plate scale we tested and optimized the influence of pH, temperature, additives, reagent volumes and reaction times (data not shown).

In the resulting method, 48 culture supernatants are filled in a 384-well plate in triplicates both undiluted as well as in one dilution step. Moreover, specific standards for the amino acid of interest are prepared by the liquid handling station from a stock solution in 9 replicates per standard on the same 384-well plate. The reaction is subsequently initiated by the addition of the Ninhydrin solution, incubated at room temperature and stopped after 4 minutes by the addition of water (see Methods section for more details). Finally, the plate is automatically transferred to the photometer integrated on the MPP and the formation of the Ruhemann’s purple is quantified at 570 nm.

Noteworthy, when measuring a culture supernatant, the formed Ruhemann’s purple results from the reaction of Ninhydrin with all amino acids present in the sample and cannot be directly accounted to a single amino acid species. Moreover, it was reported that the yield of the Ninhydrin reaction is not identical among different amino acids, because the residual groups can influence conversion rates and equilibria of the reaction intermediates [7]. In order to check the sensitivity and selectivity of the developed microtiter plate approach, we first measured 19 different amino acids, each at a concentration of 10 mM. By comparing the resulting signal intensities we indeed observed great differences in the Ruhemann’s purple formation (Figure 2B). We then tested the influence of selected media components and cultivation by-products that typically play a role during the cultivation of the model amino acid producer *Corynebacterium glutamicum*. As a result, only free alpha amino groups reacted with Ninhydrin, while none of the tested media components or by-products resulted in a detectable absorption. Finally, we checked the linear dynamic range of the assay, exemplarily for the amino acid L-lysine (Figure 2C). The resulting concentration range of 1.56 to 25 mM directly covers most of the L-lysine titers typically reached by *C. glutamicum* producer strains under lab-scale screening conditions [8-10]. Therefore, large series of error-prone dilution steps can be skipped, what further improves the speed and accuracy of the microtiter plate assay.

In another approach, we established the quantification of D-glucose and the emerging carbon source D-xylose by implementing two-step enzymatic assays on the MPP (Figure 2D and E). D-glucose is first phosphorylated via Hexokinase (HK) and then oxidized by Glucose-6-phosphate dehydrogenase (G6PDH) under formation of NADH. D-xylose is first interconverted from the α-anomeric to the ß-anomeric form by D-xylose mutarotase (XMR) and then oxidized by ß-xylose dehydrogenase (ß-XDH) under NADH formation. Prior to the D-xylose assay, D-glucose was removed from the samples with HK, since a slight side activity of the ß-XDH had been reported elsewhere [11].

Both enzymatic assays were fully automated for routine application and run without any manual work, except for the preparation of specific mastermix solutions (see Methods section). For each assay 48 cultivation samples were pipetted from a DWP to a 384-well plate in triplicates of two different pre-dilution steps. Standard samples for each run were prepared by the liquid handling station from a stock solution of D-glucose or D-xylose and pipetted in 9 replicates per standard on 384-well plates. For D-xylose quantification, the aforementioned D-glucose removal was achieved by first mixing the samples with HK and ATP surplus. After 2 minutes incubation the blank at 340 nm was measured and later subtracted from the final absorbance. Subsequently, both assays were started by addition of a mastermix and incubated for 30 minutes. Finally, the plates were automatically transferred to the photometer on the MPP and measured at 340 nm. The dynamic linear ranges for the developed microtiter plate assays were 0.05 to 0.4 g l^−1^ for D-glucose and 0.025 to 0.6 g l^−1^ for D-xylose (cf. Figure 2D and E).

### Characterization of L-lysine producers on the MPP

In a first MPP application, we screened a mutant library of *C. glutamicum* L-lysine producer strains for altered growth and production performances. The strain library consisted of 17 different genome-reduced L-lysine producers (GRLP) which were recently constructed by the targeted deletion of non-essential gene clusters from the model L-lysine producer DM1933 [12]. L-lysine production is generally coupled to primary metabolism [13], thus, highest product titers are expected after turn to stationary phase in a batch culture.

**Figure 3:**
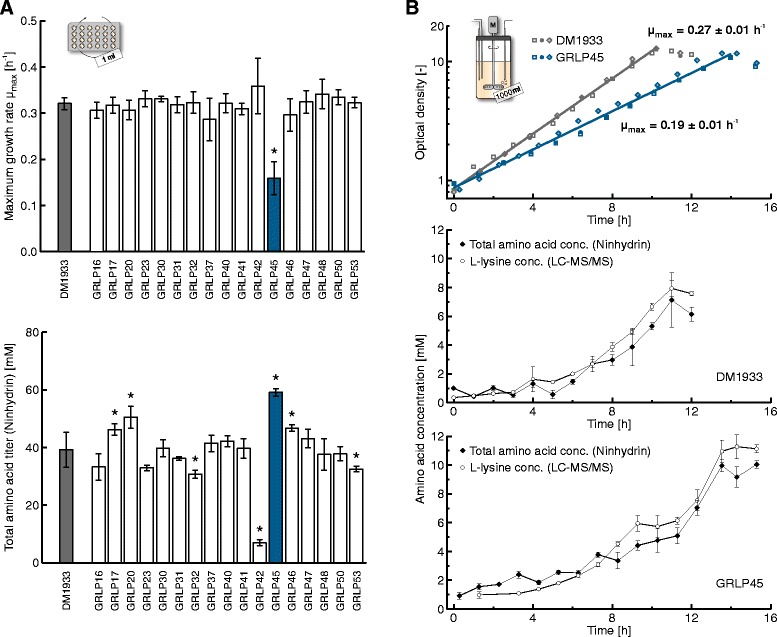
**Screening of a strain library on the MPP and scale-up of the best performer in lab-scale bioreactors. A**: Determination of maximum growth rate and total amino acid titer of a set of genome reduced L-lysine producers (GRLP) using the developed harvest procedure in combination with the Ninhydrin assay (*n* ≥ 4 biological replicates, cf. Figure 1B and 2C). Strains with significant changes in either parameter compared to the model L-lysine producer DM1933 were determined by one-way ANOVA (*p* < 0.01) and marked with an asterisk. GRLP45 showed highly elevated amino acid titers in BioLector cultivations while displaying a decreased maximum growth rate. **B**: Both observations for GRLP45 obtained on the MPP were confirmed in 1 l lab-scale bioreactor experiments (*n* = 3 biological replicates). Amino acids and L-lysine were quantified in one cultivation run of GRLP45 and DM1933, respectively, using the automated Ninhydrin assay as well as an established LC-MS/MS protocol (*n* = 3 technical replicates).

DM1933 and all 17 GRLP were cultivated multiple times (n ≥ 4) in CGXII medium with 40 g l^−1^ D-glucose on the MPP. The developed harvest procedure was used to automatically generate and freeze cell-free supernatants from all cultures, one hour after turn to stationary phase (cf. Figure 1B). Subsequently, the DWP with all frozen samples was thawn to measure the total amino acid titers by following the automated procedure described above. As a result, GRLP45 showed the lowest maximum growth rate (μ_max_ = 0.16 ± 0.04 h^−1^) which was only half the rate of the reference strain DM1933 (μ_max_ = 0.32 ± 0.01 h^−1^) (Figure 3A). In contrast, the highest amino acid titers were measured in the supernatants of GRLP45 (c_AA,max_ = 59.1 ± 1.3 mM) while the reference strain DM1933 produced only two thirds of GRLP45 (c_AA,max_ = 39.2 ± 6.1 mM).

In further experiments we tested whether the screening results obtained on our MPP can be transferred to lab-scale bioreactors. Therefore, GRLP45 and DM1933 were cultivated in 1 l bioreactors on CGXII medium with 10 g l^−1^ D-glucose (Figure 3B). Samples were taken manually every hour and subsequently measured with the developed Ninhydrin assay for total amino acid concentration as well as with an established LC-MS/MS protocol for L-lysine concentration. As a result, the significantly slower growth of GRLP45 (μ_max_ = 0.19 ± 0.01 h^−1^) compared to DM1933 (μ_max_ = 0.27 ± 0.01 h^−1^) was reproduced in bioreactor scale. Moreover, the Ninhydrin assay indicated once more a higher total amino acid production by GRLP45 compared to DM1933, which was confirmed to be an increased L-lysine titer by the LC-MS/MS measurements. Noteworthy, the highest amino acid and L-lysine concentrations were observed one hour after turn to stationary phase in all bioreactor cultivations, basically supporting the set-up of the harvest procedure for this particular product on the MPP.

### Fast assessment of substrate uptake kinetics on the MPP

In another proof of principle study we determined substrate uptake kinetics of the newly constructed strain *C. glutamicum xylXABCD*_*Cc*_*.* This strain contains a pEKEx3-plasmid with five genes for D-xylose assimilation via the Weimberg pathway to enable the growth of *C. glutamicum* on D-xylose with minimized carbon loss [14]. In general, the phenotypic characterization of such a newly constructed strain would typically start with a shaking flask or bioreactor experiment followed by a successive sampling to determine the specific uptake rates for the new carbon substrate. However, with the developed methods at hand we now can address this question in a fully automated manner on our MPP.

46 wells of one 48-well FlowerPlate were filled with an identically inoculated culture of *C. glutamicum xylXABCD*_*Cc*_ on CGXII medium with 10 g l^−1^ D-glucose and 30 g l^−1^ D-xylose. Two wells were only filled with CGXII medium and carried as sterile controls. At the start of the cultivation the first well was instantly harvested and the cell-free supernatant was frozen as first sample. The remaining wells were harvested one by one following a time-dependent pattern after the backscatter had surpassed a threshold value (cf. Figure 1C). After the cultivation was finished, all frozen cell-free supernatants were thawed to quantify the remaining D-glucose and D-xylose concentrations by following the automated procedures described above. In addition, both substrates were quantified via HPLC measurement to validate the results with an established method.

**Figure 4:**
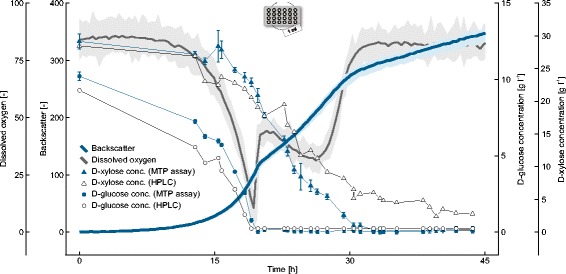
**Substrate uptake characteristics of** ***C. glutamicum xylXABCD***_***Cc***_ **during growth on CGXII medium with D-glucose and D-xylose.** The cultivation was performed in 46 identically inoculated wells of a FlowerPlate, of which each well was harvested automatically following a time-dependent pattern (cf. Figure 1C). Supernatant samples were automatically clarified via centrifugation, stored at −4°C and subsequently analyzed for D-glucose and D-xylose concentrations (*n* = 3 technical replicates). The results of both enzymatic substrate quantification methods were confirmed with established HPLC protocols. Mean values for backscatter and dissolved oxygen were estimated from unsampled replicate cultures (*n* ≥ 3). Confidence intervals (shaded areas) were spanned from the minimum and maximum value at each measurement point.

The phenotype of all parallel cultivated cultures was highly comparable (Figure 4) and, until the first harvest at *t* = 13 h, the well-to-well variability (coefficient of variation) between all 46 cultures was estimated as 3.9% and 4.4% for the backscatter and dissolved oxygen measurements, respectively. The averaged dissolved oxygen (DO) signal indicated a short interruption of oxygen consumption after 20 hours, before the DO dropped again until 26 hours. This dynamic pattern was accompanied by a drop in the maximum growth rate of the culture from μ_max,I_ = 0.28 ± 0.01 h^−1^ to μ_max,II_ = 0.08 ± 0.01 h^−1^. Both observations already pointed to a bi-phasic growth behavior which could subsequently be confirmed by the substrate analytics established on the MPP. As shown in Figure 4, within a first growth phase mainly D-glucose was utilized as carbon source while the pronounced catabolization of D-xylose first started after D-glucose becomes limiting.

All D-glucose concentrations determined with the microtiter plate assay are in good agreement with the measurements obtained by the established HPLC protocol. However, in case of D-xylose the concentration data only matched for the first samples. Noteworthy, the complete consumption of D-xylose was observed after 31 hours by the microtiter plate assay, which is also in accordance with the rising DO signal and a second bend in the backscatter curve. At this time the HPLC measurements indicated a higher remaining D-xylose concentration (5.2 mM) and these deviations were most likely caused by the limited selectivity of the applied HPLC method. Indeed subsequent GC-ToF-MS measurements of all supernatants confirmed the presence of significant amounts of D-xylonate as a by-product from D-xylose assimilation via the Weimberg pathway [14], and both compounds can actually not be separated by HPLC [15]. This exemplary result shows that the developed microtiter plate assays are not only faster, but can also lead to better separation results as compared to standard HPLC approaches, providing that the specificity of the applied enzyme for its substrate is high enough.

## Conclusions

In a previous work we showed how automated sampling with liquid handling robotics can improve the reproducibility of cultivation experiments in microtiter plates [4]. In this work we report the further improvement of our robotic Mini Pilot Plant (MPP) by introducing rapid clarification of supernatant samples and quantification of metabolites in microtiter plate scale. We show the application of completely autonomous workflows to generate cell-free supernatants from microtiter plate cultivations within minutes.

To quantify metabolites in samples at elevated throughput we developed biochemical and enzymatic assays on our MPP. The well-established Ninhydrin reaction for amino acid quantification was transferred from manual and time-consuming milliliter-scale protocols to a fast and parallelized 384-well microtiter plate scale application. Recently, the Ninhydrin assay was successfully applied to screen 311 bacterial isolates for those growing on 1-aminocyclopropane-1-carboxylate as sole carbon source [16]. However, in this study all cultures were harvested and clarified manually and the Ninhydrin assay was carried out in open 96-well plates in a boiling water bath by following single pipetting steps. In contrast, our Ninhydrin assay is operated in 384-well plates and omits heating and sealing steps, which greatly minimizes the assay duration and simplifies the automation. By the usage of DMSO we observed reduced precipitation of the Ruhemann’s purple as also reported elsewhere [17]. With this increased color stability we observed a linear detection range of 1 to 25 mM for the particular amino acid L-lysine. As product titers produced by *C. glutamicum* are typically found in this range, the assay provides the benefit to estimate the total amino acid concentration in undiluted cultivation samples within minutes. In a MPP screening with our improved Ninhydrin assay, we found the *C. glutamicum* strain GRLP45 to produce higher L-lysine titers as compared to DM1933, which was subsequently confirmed in lab-scale bioreactor experiments. The reason for the increased product formation of GRLP45, harboring deletions in the gene cluster Δ2990-3006 [12], is currently under investigation.

In further work, we focused on the substrate side and transferred enzymatic assays for D-glucose and D-xylose to 384-well plate scale to establish their quantification on the MPP. All dilutions of standards and samples were executed by the liquid handling station to achieve fast and highly reproducible results. In summary, 48 cultivation samples are automatically processed and measured in triplicates of two different dilutions within less than 30 minutes. The throughput of our method is less than one minute per sample measured in replicates and, hence, competitive to other quantitative methods based on HPLC or MS technologies. Moreover, the costs per sample for chemicals and consumables are low and robotic workstations can work with low demand on maintenance. To apply our assays, we quantified D-glucose and D-xylose in cell-free supernatants derived from a time-dependent sampling of batch cultures with the newly constructed strain *C. glutamicum xylXABCD*_*Cc*_ [14]. From the completely automated workflow of cultivation, harvest and substrate quantification, we could deduce that *xylXABCD*_*Cc*_ grows in two phases on D-xylose/D-glucose substrate mixtures and switches to D-xylose consumption when D-glucose has been depleted. The fast assessment of such phenotypes at microtiter plate scale omits the more time-demanding bioreactor cultivations for this task. Consequently, it builds a solid basis for subsequent *in-depth* phenotyping experiments including quantitative omics measurements requiring at least milliliter scale operations.

Recently, Knepper et al. reported on robotic workflows for intermittent measurement of OD, pH and metabolites from cultures in 96-well plates in seven cycles at fixed times during 48 h cultivations [18]. Their successive sampling from plates incubated without humidity control led to a total volume reduction of 47% per well over 48 hours. For product analysis, cultures were harvested and clarified manually after cultivation and at-line enzymatic substrate analytics were performed without biomass separation resulting in incorrect measurement values for D-glucose concentration. In contrast, our BioLector-based system shows lower evaporation (<10% per well over 48 hours) and, most importantly, we avoid repeated sampling from identical wells to minimize disturbance of the culture, i.e. by altering oxygen transfer due to changing culture volumes [6]. Moreover, the established workflow allows to initiate sampling events in response to individually measured online biomass values and the fully automated substrate and product assays run with cell-free samples in 384-well plates with an accordingly higher number of technical replicates.

In summary, the presented MPP allows a quick assessment of questions typically encountered during early-stage bioprocess development, i.e. during a strain screening or the first quantitative phenotyping of a few selected strains. The bottleneck of sample analytics from parallel microliter scale cultivations must be tackled nowadays and could be solved by applying easily adaptable microtiter plate assays in combination with robotic automation.

## Methods

If not stated otherwise, all chemicals or consumables for liquid handling used in this study were purchased from SIGMA Aldrich, Carl Roth or Greiner Bio-One, respectively.

### Growth medium

Cultivations were performed on defined CGXII medium which contained per liter of distilled water: 20 g (NH_4_)_2_SO_4_, 1 g K_2_HPO_4_, 1 g KH_2_PO_4_, 13.25 mg CaCl_2_*2H_2_O, 0.25 g MgSO_4_*7H_2_O, 1 mg FeSO_4_*7H_2_O, 1 mg MnSO_4_*H_2_O, 0.02 mg NiCl_2_*6H_2_O, 0.313 mg CuSO_4_*5H_2_O, 1 mg ZnSO_4_*7H_2_O, 0.2 mg biotin and 30 mg protocatechuic acid [19]. The primary carbon and energy source was D-glucose with 10 or 40 g l^−1^ or a mixture of 10 g l^−1^ D-glucose with 30 g l^−1^ D-xylose. The medium for cultivation in bioreactors or microtiter plates was supplemented with 3% (v v^−1^) AF204 antifoam agent or 5 g l^−1^ urea and 42 g l^−1^ MOPS buffer, respectively. For medium preparation some substances were added sterile after autoclaving (D-glucose/D-xylose, PCA, biotin, trace elements and AF204) and 4 M HCl/4 M NaOH was used to adjust the pH to 7.0 in bioreactors.

### Strain storage and cultivation

Cryo cultures of all strains were prepared from exponentially growing cultures on CGXII medium in shaking flask. Cells were harvested at OD_600_ = 10, washed once with 0.9% (w v^−1^) NaCl and stored at −80°C in a solution containing 20% (v v^−1^) glycerol and 0.9% (w v^−1^) NaCl.

Bioreactor cultivations were carried out in 1.5 l reactors (DASGIP AG, Jülich, Germany) in batch mode at 30°C and 1 vvm air flow. Aerobic process conditions were controlled via stirrer speed (200 – 1200 rpm) to maintain 30% dissolved oxygen (DO) concentration. The pH of the culture was regulated to pH 7.0 with 4 M HCl and 4 M NaOH. Online measurements were taken for pH (405-DPAS-SC-K80/225, Mettler Toledo) and DO (Visiferm DO 225, Hamilton). Cultures were inoculated per liter with 0.5 ml cryo culture aliquots and sampling as well as monitoring of growth was started when cultures had reached an OD > 0.5 on the next day. Samples for biomass determination were taken every hour and measured photometrically at λ = 600 nm (OD_600_).

Microtiter plate cultivations were carried out in 48-well FlowerPlates (m2p-labs GmbH, Baesweiler, Germany) with DO and pH optodes in a BioLector (m2p-labs GmbH) at 1000 rpm, 95% humidity, 30°C and backscatter gain 20. Cultures were started at OD_600_ = 0.1 by inoculation of 990 μl medium with 10 μl cryo culture per well. Maximum growth rates were calculated directly from backscatter values as described elsewhere [12].

### Robotic workflow of automated harvest

Robotic workflows were developed on a JANUS Automated Workstation (PerkinElmer, Waltham MA, USA) equipped with a pipetting arm (Varispan) with 8 steel needles and a gripper arm for transport of plates. The track of both arms was extended by 400 mm in order to reach a BioLector (m2p-labs, Baesweiler, Germany), the MTP centrifuge IXION (Sias, Hombrechtikon, Switzerland) and the MTP photometer EnSpire (PerkinElmer, Waltham MA, USA) outside of the regular liquid handling deck. Cooling of samples down to −10°C was performed on a DWP cooling rack (MeCour, Groveland MA, USA) connected to the cryostat Unichiller (Huber, Offenburg, Germany). Further details about the setup and evaluation of the robotic workstation were described elsewhere [4].

Cultivations for automated harvesting were performed with 22 minute measurement cycles in the BioLector and monitored by the RoboLector agent software (m2p-labs, Baesweiler, Germany). This software pauses as well as opens the BioLector and writes a CSV-based handshake file, provided that trigger conditions (here: timer after biomass threshold) had been reached. This handshake file indicates positions of those wells of the FlowerPlate that have reached their individual trigger condition and was then immediately used by the liquid handling platform to transfer 500 μl of those wells from the BioLector to a DWP. Subsequently, the handshake file was first copied by automated execution of a batch script, before the original file was deleted by the WinPrep software (Version 4.6, PerkinElmer). This deletion process triggers the RoboLector agent software to close the BioLector lid and to continue the cultivation. The copied handshake file was then used by the liquid handling platform to fill a second DWP with water as tare. Both DWPs were transferred to the IXION centrifuge and rotated at 4500 rpm for 5 minutes. The obtained cell-free supernatants were aspirated completely at a fixed height (4 mm above well bottom) and transferred to a third DWP at −4°C, closed with an aluminum sealing foil. Finally, the tare plate was also emptied at the same height to equal its weight for the following centrifugation steps. The developed protocol takes 12 to 17.5 minutes depending on the number of wells harvested at a time and runs without manual intervention or replacement of consumables (DWPs). The resulting cell-free supernatants were stored in the −4°C DWP in the same layout as in the BioLector experiment in order to avoid cross contamination.

### MTP Ninhydrin assay

As Ninhydrin solution for the MTP assay 2 g Ninhydrin were solved in 75 ml DMSO and 25 ml 4 M sodium acetate buffer (pH 6.0 adjusted with 25% acetic acid). The mastermix was prepared fresh for each experiment and stored in dark until use. A 100 mM L-lysine-HCl stock solution was prepared in CGXII medium for standard dilution series.

The robotic workflow of the Ninhydrin assay was started with three independent 1:2 dilution series of the L-lysine stock solution using CGXII medium down to 1.56 mM in a DWP. 30 μl of each dilution series was pipetted in triplicates in a 384-well plate, resulting in a total number of 9 wells for each standard concentration. 48 cell-free supernatants were pipetted from a DWP to the 384-well plate in triplicates of 45 μl. Subsequently, 15 μl of these undiluted samples were aspirated from the 384-well plate and dispensed in other wells of the same plate and mixed with 15 μl medium to derive 1:2 dilutions of each cultivation sample in triplicates. The reaction was finally started with addition of 30 μl Ninhydrin solution to each well, incubated at room temperature and stopped after exactly 4 minutes by the addition of 40 μl distilled water. The plate was immediately transferred to the photometer on the MPP by the gripper arm and read for absorbance at λ = 570 nm. During subsequent analysis, raw data were first blanked by wells with sole medium as sample, before the standard curve (1.56 – 25 mM) was used to quantify total amino acids in the cell-free supernatants.

### MTP assay for D-glucose and D-xylose quantification

Enzymatic reactions were used to quantify D-glucose and D-xylose in cell-free supernatants on the MPP. For the D-glucose assay a mastermix was freshly prepared by mixing: 47.4 ml TRIS-maleat buffer (100 mM, pH 6.8), 2.2 ml MgSO_4_ solution (100 mM), 1 ml NAD^+^ stock (50 mg ml^−1^), 1 ml ATP stock (34 mg ml^−1^) and 236 μl Hexokinase/Glucose-6-phospate dehydrogenase mix (Roche Diagnostics, Mannheim, Germany). The automated workflow on the MPP started with the preparation of three independent dilution series of standards in the range of 0.4 to 0.01 g l^−1^ from a 1 g l^−1^ D-glucose stock. Afterwards, 20 μl of each standard solution was pipetted in triplicates in a 384-well plate, resulting in 9 wells for each D-glucose standard concentration. All 48 cell-free supernatants were diluted in two different steps (typically 1:10 and 1:40) by successive aspiration of water and sample, followed by common dispense in a DWP. 20 μl of these 96 diluted samples were subsequently pipetted in a 384-well plate in triplicates. Then, 80 μl mastermix was added to each well before the plate was incubated for 45 minutes at room temperature to allow for a complete reaction. Finally, the plate was automatically transferred to the photometer and measured for absorbance at λ = 340 nm. During analysis, raw data were first normalized by wells with water as sample, before the standard curve (0.01 – 0.4 g l^−1^) was used to quantify D-glucose in the cell-free supernatants.

For the D-xylose assay two mastermixes were prepared, the first (M1) by mixing: 47.4 ml TRIS-maleat buffer (100 mM, pH 6.8), 2.2 ml MgSO_4_ solution (100 mM), 1 ml NAD^+^ stock (50 mg ml^−1^), 1 ml ATP stock (34 mg ml^−1^) and 236 μl Hexokinase (Megazyme, Wickow Ireland). For the second mastermix (M2) 5.09 ml distilled water was mixed with 410 μl β-xylose-Dehydrogenase/D-xylose mutarotase mix (Megazyme, Wickow Ireland). The automated workflow started with the preparation of standards in three independent dilution series ranging from 0.6 to 0.01 g l^−1^ from a 1.5 g l^−1^ D-xylose stock. Afterwards, 10 μl of each standard solution was pipetted in a 384-well plate in triplicates, resulting in 9 wells for each D-xylose standard concentration. For the D-xylose assay all 48 samples were diluted 1:10 and 1:100 to cover the whole expected range of D-xylose concentrations and 10 μl of these diluted samples were pipetted in a 384-well plate in triplicates. Then, 80 μl of the M1 was added to each well to allow a complete removal of D-glucose in order to exclude unspecific reactions of the D-xylose mutarotase. After 2 minutes incubation at 37°C the absorbance at λ = 340 nm was measured before 10 μl of the M2 was added to all wells. Afterwards, the plate was again incubated at 37°C for 30 minutes until the final absorbance at λ = 340 nm was measured. During data analysis, the difference between absorbance after first reaction and second reaction was calculated in order to blank the final values with the background absorbance after the enzymatic removal of D-glucose.

### LC-MS/MS and HPLC measurements

For LC-MS/MS analysis, cell-free supernatants were first pre-diluted with distilled water to the linear range (0.025 μM – 25 μM) of the LC-MS/MS protocol. To eliminate artefacts from the running buffer and the used internal standard the last 1:2 dilution step was performed with 100% MeOH to gain a final 50% MeOH concentration in the sample. Targeted quantification of L-lysine in supernatant samples was conducted by isotope dilution mass spectrometry as described by Paczia et al. [20].

D-glucose and D-xylose were quantified by HPLC with a 300 × 8 mm organic acid column (CS Chromatographie, Langerwehe, Germany) at 40°C using isocratic elution with 0.1 M H_2_SO_4_ at a flow rate of 0.5 ml min^−1^. Carbohydrates were detected via refraction index (Agilent, Santa Clara, CA, USA) and concentrations were determined by calibration with external standards.
